# Microbial associates of an endemic Mediterranean seagrass enhance the access of the host and the surrounding seawater to inorganic nitrogen under ocean acidification

**DOI:** 10.1038/s41598-023-47126-4

**Published:** 2023-11-15

**Authors:** Catherine A. Pfister, Ulisse Cardini, Alice Mirasole, Luis M. Montilla, Iva Veseli, Jean-Pierre Gattuso, Nuria Teixido

**Affiliations:** 1 The Department of Ecology and Evolution, The University of Chicago, Chicago, IL USA; 2 Integrative Marine Ecology Department, Stazione Zoologica Anton Dohrn - National Institute of Marine Biology, Ecology and Biotechnology, Naples, Italy; 3 Biophysical Sciences Program, The University of Chicago, Chicago, IL USA; 4 CNRS, Laboratoire d’Océanographie de Villefranche, Sorbonne Université, 181 Chemin du Lazaret, 06230 Villefranche-sur-Mer, France; 5 Institute for Sustainable Development and International Relations, Sciences Po, 27 Rue Saint Guillaume, 75007 Paris, France

**Keywords:** Ecosystem ecology, Microbial ecology, Marine biology, Element cycles

## Abstract

Seagrasses are important primary producers in oceans worldwide. They live in shallow coastal waters that are experiencing carbon dioxide enrichment and ocean acidification. *Posidonia oceanica*, an endemic seagrass species that dominates the Mediterranean Sea, achieves high abundances in seawater with relatively low concentrations of dissolved inorganic nitrogen. Here we tested whether microbial metabolisms associated with *P. oceanica* and surrounding seawater enhance seagrass access to nitrogen. Using stable isotope enrichments of intact seagrass with amino acids, we showed that ammonification by free-living and seagrass-associated microbes produce ammonium that is likely used by seagrass and surrounding particulate organic matter. Metagenomic analysis of the epiphytic biofilm on the blades and rhizomes support the ubiquity of microbial ammonification genes in this system. Further, we leveraged the presence of natural carbon dioxide vents and show that the presence of *P. oceanica* enhanced the uptake of nitrogen by water column particulate organic matter, increasing carbon fixation by a factor of 8.6–17.4 with the greatest effect at CO_2_ vent sites. However, microbial ammonification was reduced at lower pH, suggesting that future ocean climate change will compromise this microbial process. Thus, the seagrass holobiont enhances water column productivity, even in the context of ocean acidification.

## Introduction

Associations between marine species and microbes have been increasingly identified with genomic methodologies, revealing many hidden species interactions between eukaryotic and prokaryotic partners^1–3^. In addition to affecting host fitness, these interactions may affect global biogeochemical cycles, through the use and cycling of carbon, nitrogen and other elements^4,5^. Seagrasses are marine angiosperms and are foundational species in coastal ecosystems that are critical in structuring these ecosystems and modulating the flux of nutrients and energy. They provide habitat for other species^6,7^, are key primary producers^8,9^, and also act as carbon sinks by sequestering carbon^10,11^. Marine angiosperms are some of the most widely distributed plants on the planet^12^, and recent studies suggest they host a diversity of microbial taxa^13,14^, with some functional partnerships demonstrated^15,16^.

Among seagrasses, a number of microbial taxa have been identified as important to nutrient cycling, including microbes capable of nitrogen fixation^15^, ammonification^16^ and sulfur oxidation^13,14^. *Posidonia oceanica*, the seagrass species that dominates the Mediterranean Sea, achieves high abundances in coastal habitats with relatively low seawater dissolved inorganic nitrogen concentrations. Microbial partnerships may partly explain its productivity. First, the low oxygen environment surrounding the roots provides a microenvironment for nitrogen fixation^15,17^ and other reducing metabolisms. Second, seagrasses might have access to the dissolved organic nitrogen from the surrounding seawater. A related species of *Posidonia* in Australia has microbial associates that ammonify amino acids—resulting in dissolved inorganic nitrogen that is taken up as ammonium^16^. Although it has been claimed that seagrasses can directly take up dissolved organic nitrogen sources (e.g.^18^), we know of no studies that show this while controlling for the role of bacteria.

Bacteria that deaminate amino acids to produce ammonium are likely abundant in nature, given that there is a diversity of enzymes that cleave carbon–nitrogen bonds and produce ammonium^14^. Whether these bacteria are consistently associated with hosts is relatively little studied, including what determines their abundance. Studies that focus only on the taxonomy of microbes (e.g. amplicon sequencing) may not reveal the functional capacity of microbes^19^. Information on amino acids in seawater in the environments where marine macrophytes are abundant are also lacking and infrequently quantified. Nonetheless, dissolved organic compounds can be abundant in coastal waters and can be a key part of nutrient recycling^20^.

While the discovery of host-microbe interactions is still nascent, populations of many marine macrophytes are in decline^6,21,22^. For seagrasses, global environmental change, including ocean acidification, warming, pollution, and physical disturbances such as increased turbidity represent key threats^23^. Future ocean conditions will include a continued decrease of pH and a change in carbonate system parameters. While marine angiosperms such as *P. oceanica* may benefit from increased bicarbonate concentrations^24^, little is known about how their associations with microbes may be altered. If increased bicarbonate access improves carbon fixation and fitness, host fitness might increase and microbial associates may benefit. If the availability of inorganic carbon increases while nitrogen remains the same, the release of excess carbon via stoichiometric overflow, could increase^25^. While this carbon release could benefit heterotrophic host-associated microbes, nitrogen limitation of microbial growth might also result. Low pH could also be a stressor to the host and host health, with potential consequences for the diversity and/or functional roles of host-associated microbes. The seagrass *Cymodocea nodosa* showed taxonomic differences in rhizome microbes but not leaf microbes across a pH gradient driven by the natural release of carbon dioxide (CO_2_) at vents in the Mediterranean^26^, though the functional differences were unexplored. The mechanisms underlying when microbes are resistant or sensitive to changes in host biogeochemistry from ocean acidification needs further understanding.

At CO_2_ vent sites in the Mediterranean the seagrass *P. oceanica* shows a number of differences compared to areas without the influence of venting CO_2_. Benthic communities near vents show a decline in calcified species^27,28^, and accordingly, calcareous epiphytes of *P. oceanica* are much reduced in proximity to the vents^8,29^. Additionally, herbivorous fish are more abundant near the vents^30^. In proximity to the vents, *P. oceanica* has a shorter stature, fewer epiphytes, increased tissue nitrogen content^31^, and increased shoot density^29,32^. Proximity to vents can increase *P. oceanica* productivity at the scale of individual leaves, but shows variable results at the community level^8^. Seawater pH is variable in the areas surrounding CO_2_ vent sites and *P. oceanica* is often in pH conditions around 7.7^28^.

The foundational role that *P. oceanica* plays in the Mediterranean^30^, its economic importance^33^, and concern about its health status^34^ makes this species important for investigating host-associated microbial function in a global change context, particularly under ocean acidification. We thus investigated microbial function at natural CO_2_ venting areas and reference areas with no venting and ambient pH. We focused on nitrogen dynamics, a relatively little studied aspect of ocean acidification (but see^35^). Populations of *P. oceanica* may have spent generations in association with decreased pH, allowing tests of community interactions and functional changes, including host-microbe interactions.

Here, we quantified whether ammonification, or the ability of microbes to make dissolved organic nitrogen available as ammonium, occurred in microbes associated with *P. oceanica,* and microbes that were free-living, and whether ammonification differed in areas of low pH at CO_2_ vents. We tested carbon fixation rates, dissolved organic carbon release rates, and tissue nitrogen values as possible plant traits that differed among sites. We used metagenomics to describe the microbial taxa in association with *Posidonia* blades and roots, and probe their functions, both at a site with CO_2_ venting with low pH and at a nearby site unaffected by vent activity with ambient pH.

## Results

### The movement of ^15^N amino acids in *P. oceanica* and seawater

**Figure 1:**
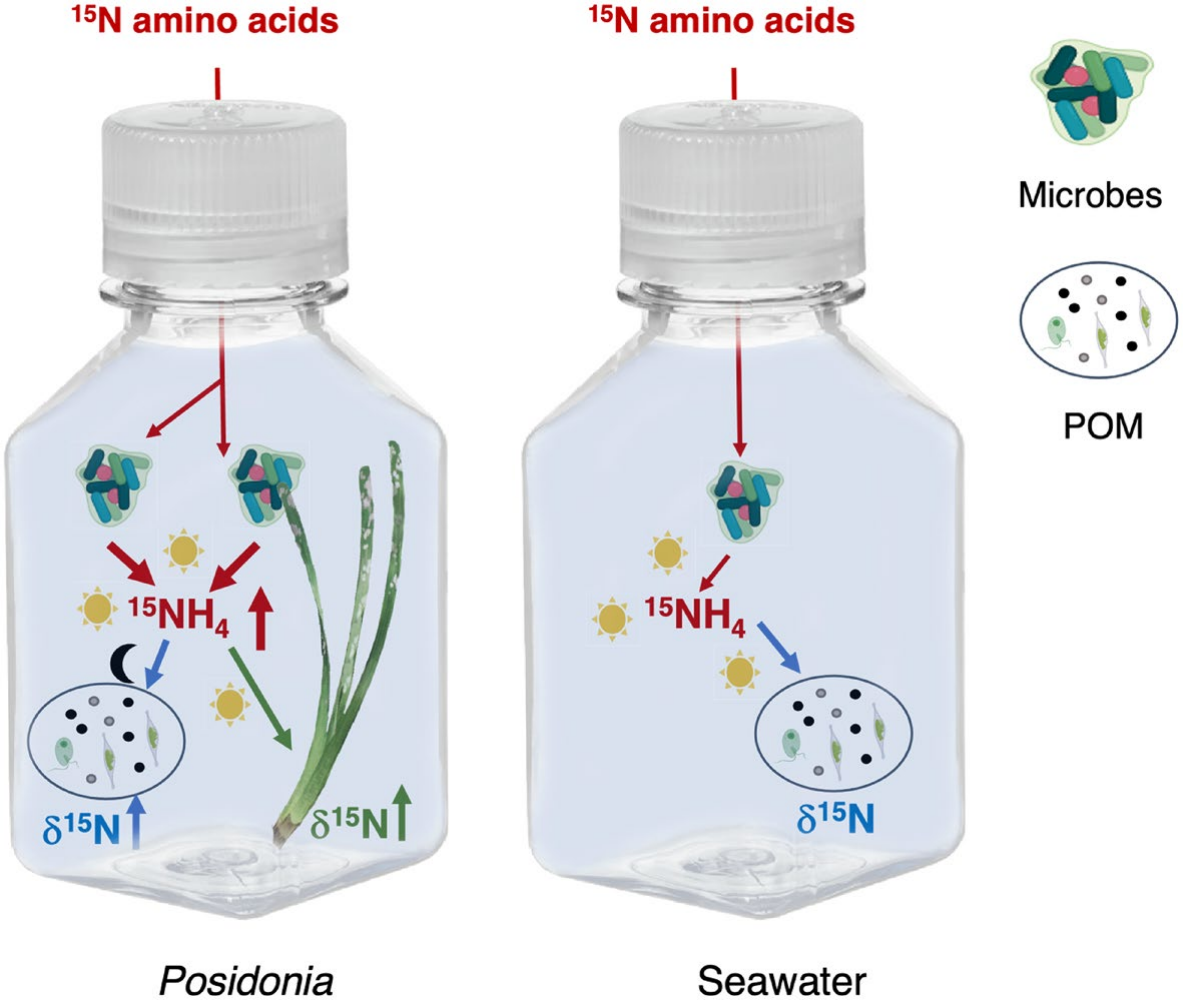
Illustration of the possible paths of δ^15^N amino acids in with the seagrass *Posidonia oceanica* or seawater only. Enlarged arrows on the left indicate processes that were greater at the control site than the CO_2_ venting site. All processes were also greater with *Posidonia* present, even if only during either the day or the night, indicated with either a sun or moon icon. All processes correspond to Fig. 2a–d and the statistical tests can be found in Table S2.

**Figure 2:**
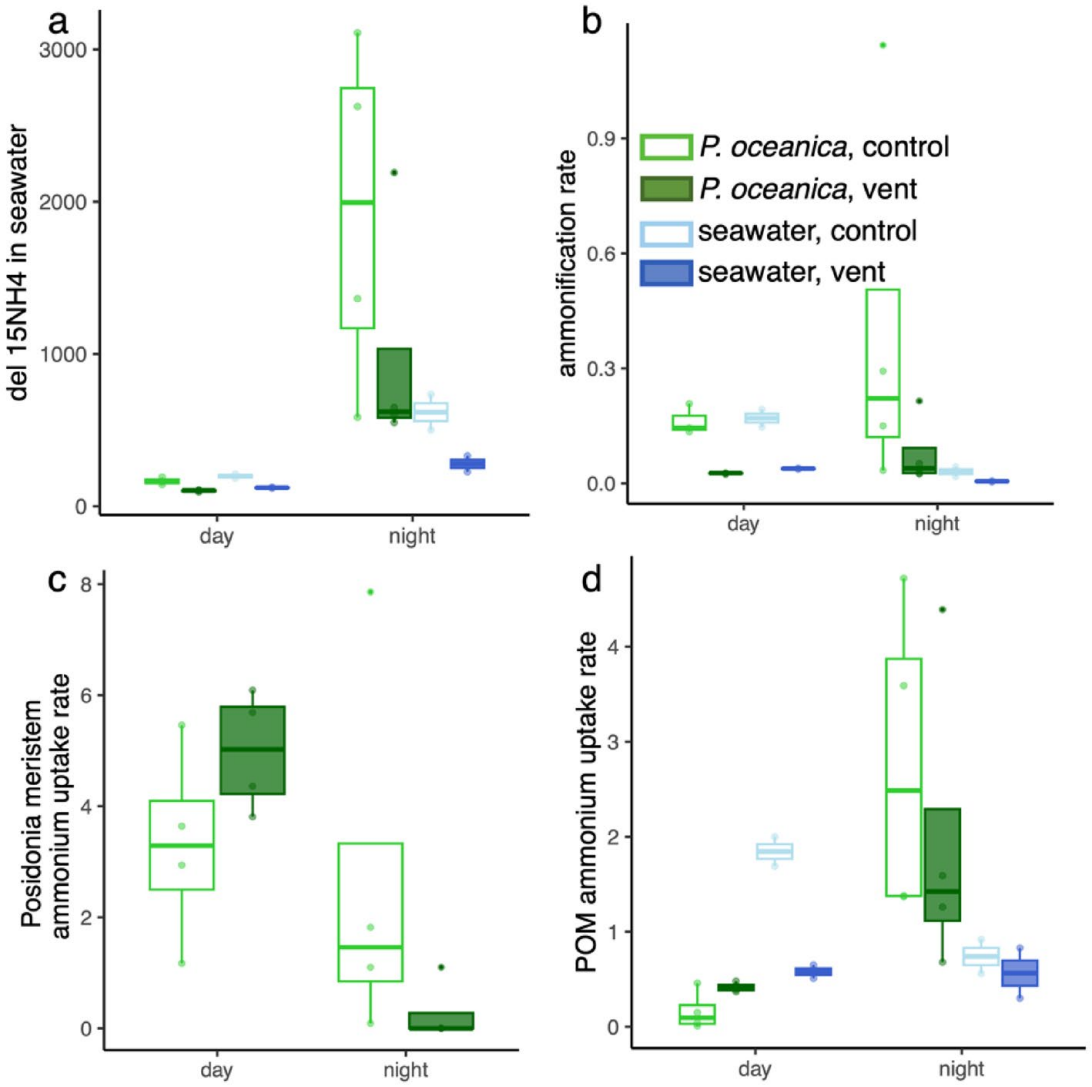
The fate of ^15^N amino acids added to incubation bottles with and without *P. oceanica*. In a, δ^15^N ammonium (‰) in seawater in incubation bottles with *P. oceanica* or seawater only. A comparison of the values during the day and night, showed a significant increase in the bottles at the end of the night, with increased values at control site and with *P. oceanica*. There was also an interaction between time and treatment; the increase at night was highest with *P. oceanica*. In b. The ammonification rate in nmol per hour across the two sites and attributed to *P. oceanica* and seawater versus seawater only based on Eq. (1). The control site had greater ammonification compared with the vent site (*p* = 0.002) and *P. oceanica* was nearly associated with greater ammonification rates than seawater (*p* = 0.060), while rates did not differ overall between night and day (*p* = 0.732). Examining *P. oceanica* separate from seawater during day versus night, *P.oceanica* has no diel pattern, while the POM in seawater is associated with greater ammonification during the day. In c, ammonium uptake rate in umol N per g dry mass per hour in the *Posidonia* meristem during both day and night periods using Eq. (2). Uptake was greater during the day (*p* = 0.002), with a significant interaction (*p* = 0.020), indicating a relatively greater uptake of ^15^N ammonium at night in the control site. In d., the uptake of ammonium by POM was greater when *P. oceanica* was absent from the incubation bottles during the day, and this effect was greatest at the control site, based on the interaction between site and treatment (*p* = 0.033). At night, the situation is reversed and the incubation bottles with *P. oceanica* resulted in a greater incorporation of ^15^N into the POM in the seawater compared with bottles that had no *P. oceanica* (*p* = 0.013). All values were log transformed prior to statistical analyses shown in Table S2.

There were differences in the fate of the ^15^N amino acids that were incubated with *P. oceanica* versus seawater only (Fig. 1), with enrichment of seawater ammonium greatest at the control site and greatest when in association with *P. oceanica* and at night (Fig. 2a). The ^15^N in ammonium in seawater (δ^15^N of NH_4_) increased during the first 7 daylight hours (‘day’) and increased again overnight (‘night’). Initial conditions for the incubation bottles are in Table S1; statistics and all measurements of *P. oceanica* are in Supplementary Tables 2 and 3, respectively.

The ammonification rate in nmol per hour across the two sites (Eq. 1, Methods), revealed greater ammonification rates at the control site with ambient pH compared with the vent site, both in seawater and when *P. oceanica* was present (Fig. 2b, p = 0.002). Across all treatments, ammonification rates were more than 4 times greater at control sites. There was a trend for greater ammonification with *P. oceanica* compared with seawater (*p* = 0.045), and ammonification with seagrass was 2.5× greater when averaged across both sites. Rates did not differ between night and day (*p* = 0.512). Comparing the ammonification rates where *P. oceanica* was in the incubation bottles, there was no diel pattern, while incubations with seawater only were associated with greater ammonification by particulate organic matter (POM) during the day (*p* = 0.002).

The very newest *Posidonia* tissue growth in the meristem showed almost 3× greater uptake of ^15^N-enriched ammonium during the day compared to night (*p* = 0.002, Fig. 2c), but the increase seen at the vent during the day switched at night to greater uptake in control sites (interaction between time of the experiment and site, *p* = 0.020, Fig. 2c). Rates of ammonium uptake by *Posidonia* reached up to 6–8 μmol N per g dry mass per hour based on estimates of ^15^N enrichment and using Eq. (2) (Fig. 2c, Table S3). *P. oceanica* plants also showed similar patterns of ^15^N enrichment in the midblade and on the blade under epiphytes (Table S3).

The nitrogen content in *Posidonia* tissue did not differ between the vent and control site, either in the meristem (t = 0.993, *p* = 0.360) or midblade (t = 0.517, *p* = 0.623), and averaged 2.1% in the meristem and 1.5% in the midblade region overall (Fig. S1).

**Figure 3:**
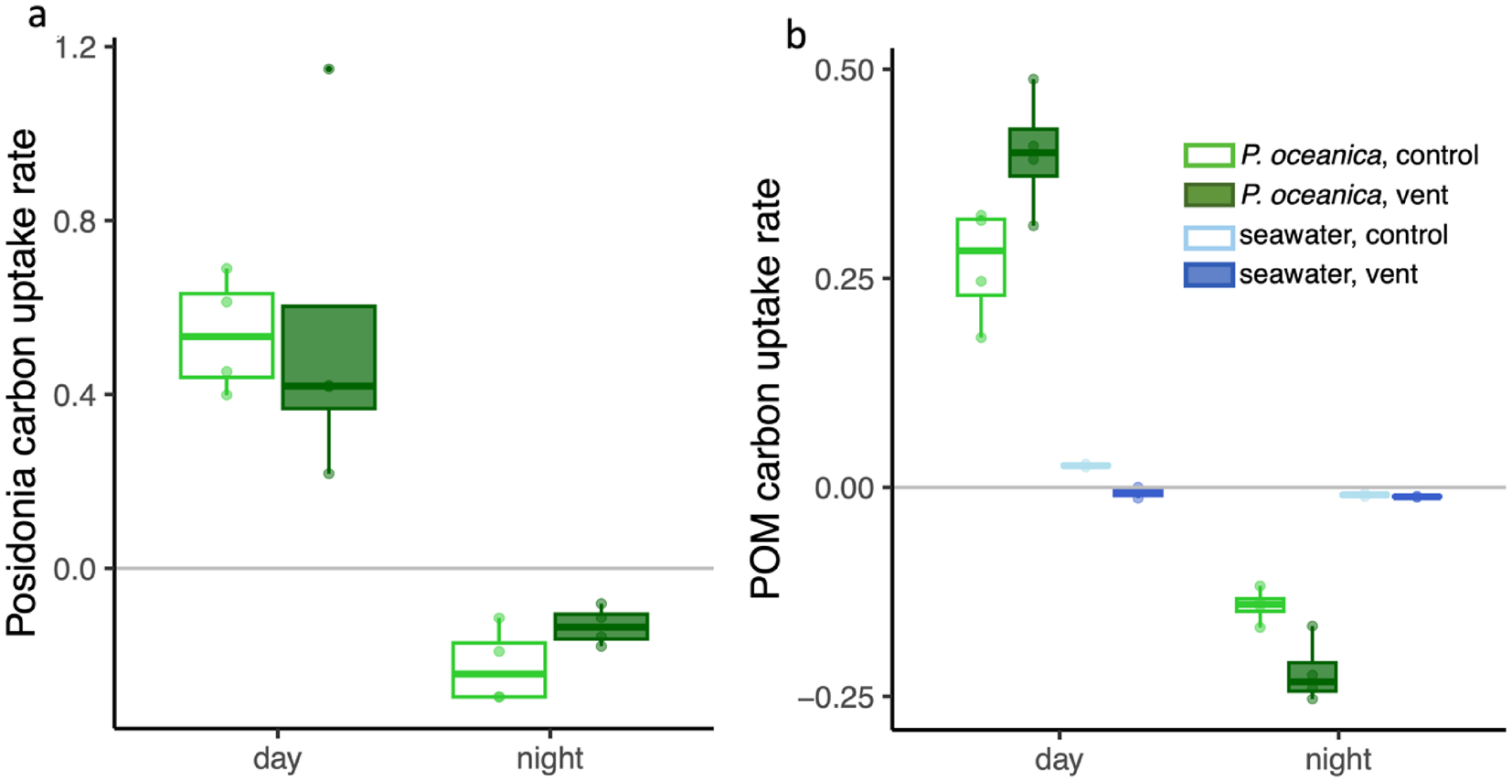
Carbon uptake rates by a. *P. oceanica* and b. seawater. In a, carbon fixation or respiration, in mg C per hour per g *P. oceanica* dry mass in incubation bottles during daylight and nighttime hours. *P. oceanica* carbon fixation was based on oxygen change, following subtraction of water column rates. *P. oceanica* carbon fixation was greater during the day, but did not differ between control and vent sites. Two-way ANOVA (site effect 0.646, diel effect *p* < 0.001, interaction *p* = 0.728). In b, carbon uptake by unfiltered seawater (in mg C per L per hour) is shown for both sites and in incubation bottles with and without *P. oceanica*. POM carbon uptake differed between day and night (p < 0.001) and with *P. oceanica* (*p* = 0.002); there were significant interactions with time of day and site (*p* = 0.004) and time of day and treatment (*p* < 0.001). Boxplots show mean values and delimit 25th and 75th percentiles; all points are shown, statistical tests in Table S2.

The daylight carbon fixation rates per unit tissue mass of *P. oceanica* did not differ whether *Posidonia* was in proximity to CO_2_ vents or in control areas (*p* = 0.646, Fig. 3a, Table S2). At night, respiration rates too were indistinguishable (Fig. 3a). The lack of difference in carbon fixation by *Posidonia* and the nitrogen content of the blades were consistent with a statistically indistinguishable C:N ratio of *P. oceanica* tissue among sites, whether we considered the meristem tissue or the midblade underneath epiphytes (Fig. S1a,b).

### Dissolved and inorganic nutrient dynamics

The dynamics of dissolved organic nutrients was affected by *P. oceanica*, proximity to CO_2_ vents, and time of day. Dissolved organic carbon (DOC) was relatively unaffected by *P. oceanica*, with a nearly significant interaction between time of day (day or night), likely reflecting the increase DOC at the vents at night (*p* = 0.017) (Fig. S2a). DON release in association with *P. oceanica* showed no difference between control and vent site as a main effect (*p* = 0.220) but there was an interaction over the diel cycle, with DON increased during the day when *P. oceanica* was fixing the most carbon (*p* = 0.003, Fig. S2b). The pattern of dissolved organic matter release in seawater only showed that DOC was greater at the vent site (*p* = 0.005) and greater at night (*p* = 0.041). DON release was also greater at the vent site, though the interaction between site and time of day (*p* < 0.001) indicated that DON release was greater during the day at the vent site and greater at night at the control site (*p* < 0.001). DOC and DON tended to have net production during the day and net uptake at night. In contrast, the use of inorganic nutrients was greater during daylight hours and is illustrated by decreased concentrations of ammonium, nitrite, nitrate and silica (Fig. S2).

### The effect of *P. oceanica* on surrounding POM

The presence of *P. oceanica* was associated with increased uptake of ^15^N in POM during nighttime hours, an effect that was greatest at the control site, based on the interaction between site and treatment (*p* = 0.001, Fig. 2d). Combining the day and night interval, POM ^15^N uptake was greater with *P. oceanica* (Table S2), resulting in 1.6 times more nitrogen uptake in chambers with *P. oceanica* compared with seawater at the control site, and 2.5× higher at the vent site.

Carbon uptake by POM was also enhanced with *P. oceanica* though only during the day; POM carbon uptake decreased with *P. oceanica* at night (Fig. 3b). The mean C:N ratio of POM was 5.09 at the control site and 5.97 at the vent site (Table S3). If the carbon:nitrogen uptake by POM was relatively constant, water column carbon uptake was increased 8.6 times (1.6 × 5.09) at the control site and 17.4 times (2.5 × 5.97) at the vent site due to the contribution of bacteria that metabolize amino acids in association with *P. oceanica*.

### Metagenome analyses

**Figure 4:**
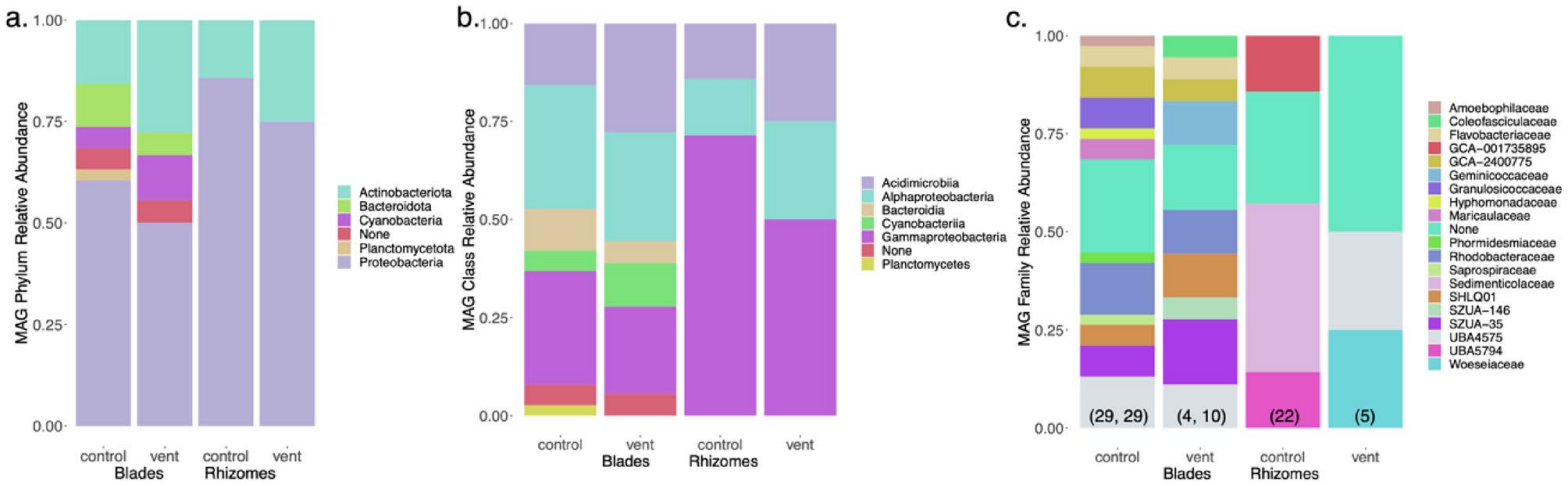
The taxonomic assignments of 67 metagenome-assembled genomes (MAGs) in the 6 samples from the blade or rhizome surface of *Posidonia* as (**a**). Phylum, (**b**). Class and (**c**). Family. There were 22 high quality MAGs and 42 medium quality MAGs, while taxonomy was not found for 3. In (**c**), alpha diversity in each sample is shown and based on ‘anvi-estimate-scg-taxonomy’. See Supplemental Table S4 and S5.

After quality control and filtering, we obtained an average of 33.7 million sequence reads per sample (range 28.6–43.8 million). Metagenomic short reads were assembled into contigs of at least 1000 nucleotides in length, resulting in 26,460–123,697 contigs per sample with a mean of 68,852 across all 6 samples (Table S4). Across all the metagenomes in our study, the taxonomy of *Posidonia* blades had the greatest alpha diversity, with rhizomes at the vent having the fewest (Fig. 4, Table S4), though due to lack of replication we are precluded from statistical tests of diversity.

The metagenomic reads allowed us to construct metagenome-assembled genomes (MAGs), ranging from only a single MAG on rhizomes at the vent site to 21 MAGs on a blade metagenome at the control site. We used Bowers et al.^36^ criteria to define a MAG as high quality if it had a competition score of > 90% and a redundancy (or contamination) of < 10% (Table S5), yielding 22 high quality MAGs. A remaining 42 were medium quality MAGs with completion scores between 42 and 90% and redundancy scores between 0 and 11% (Table S5). All MAGs were bacterial and represented 5 phyla (Fig. 4a). A notable difference among blades versus rhizomes was the presence of the Cyanobacteria and Bacteroidota phyla on blades but not on rhizomes. In three cases, we were unable to taxonomically resolve a metagenome-assembled genome. Taxonomic diversity at the class and family level was higher on blades compared with rhizomes (Fig. 4b,c).

**Figure 5:**
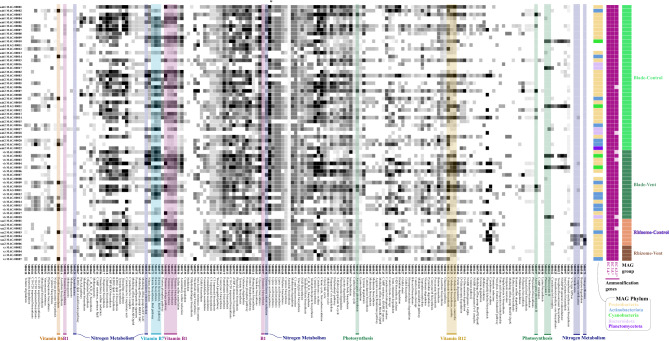
The metabolisms in 67 microbial metagenome assembled genomes (MAGs) assembled on *P. oceanica* at Ischia, Italy across 5 Phyla and on four different tissue types. The completion (black = complete, white = absent) in each microbial MAG for metabolic modules that showed differences across many of the MAGs. A full list of MAG metabolisms is in Table S7. Every MAG had some ammonification genes (Table S6). The figure was generated from anvi’o^76^.

The metabolism of these metagenomes was diverse, with some functional differences (Fig. 5). Photosynthetic and anoxygenic photosystem II were present only on blades, an expected result given the presence of Cyanobacteria only on blades. The KEGG module for nitrogen fixation was partially annotated within three MAGs, and especially the two MAGs in the genus *Thiodiazotropha*. The third MAG was an unidentified Gammaproteobacteria and was only 33% complete for nitrogen fixation genes (Table S7). Further, nitrogen fixation was indicated only on rhizome tissue and at the control site based on these three MAGs (Fig. 5, Table S7). Ammonification was a prevalent metabolism across all bacterial MAGs on *P. oceanica*. Of the 67 MAGs, all had enzymes classified as EC:1.4.* or EC:3.5.*, while 61 MAGs had enzyme function classified as EC:4.3.1* (Table S6). Nitrate reduction capability was present in the rhizome of seagrass from control areas (MAG 1 and MAG4), including denitrification in control rhizomes (MAG4).

Other microbial metabolisms that could be beneficial to *P. oceanica* included the presence of B vitamin metabolism on both tissue types at both vent and control areas. Vitamin B6 (pyridoxal) biosynthesis was only present in control rhizomes, while thiamin biosynthesis, vitamin B1, was present in all samples except vent rhizomes and vitamin B7 (biotin) biosynthesis was in multiple MAGS in both tissue types and sites. Vitamin B12 (cobalamin) synthesis was suggested in multiple MAGs at both sites, with aerobic biosynthesis only on the blades and anaerobic biosynthesis suggested in MAGs from all samples. Multiple MAGs on blades and rhizomes at both sites had sulfur oxidizing capability.

## Discussion

Proximity to CO_2_ vents changed microbial processes in association with the seagrass *P. oceanica*, reducing rates of ammonification. Carbon and nitrogen uptake by POM was also increased at the vent sites. Thus, water column processes differed between the control and vent area, possibly due to microbial nitrogen processing in association with seagrass. Despite differences in plant morphology^29,32^ and grazing pressure^30^ at vent sites, we measured similar rates of carbon fixation by *P. oceanica*.

Benthic-pelagic coupling and enhancement of pelagic productivity through the host-associated microbes on *P. oceanica* have implications due to global declines in seagrass cover^37^ and the prevalence of seagrass wasting diseases^38^. Marine macrophytes have increasingly been shown to host a species rich microbiome^39^, with diverse functions that include not only ammonification, but also nitrogen fixation^8,35,40^, nitrification^41^ and nitrate reduction^14,42^. Seagrasses are important to carbon sequestration due to carbon fixation^43^ and the carbon storage in the root phyllosphere^44^ and sediment. Seagrasses also ameliorate pH decreases^45^. Here we show that *P. oceanica* enhanced carbon fixation in the water column by over eightfold at the control site and over 17-fold at the vent site, suggesting that the contribution of seagrass to the carbon cycle is likely greater than previously recognized due to water column effects. The presence of seagrass also enhanced water column nitrogen uptake to 2.5 times at the vent site and 1.6 times at the ambient site when we integrated over both day and night intervals (Fig. 2d). A possible reason for enhanced POM carbon and nitrogen uptake is the increased dissolved organics available to heterotrophic bacteria when *P. oceanica* is present (Fig. S2). Nitrogen cycling in association with *P. oceanica* has been shown previously to be elevated near vents^35^. *P. oceanica* mirrors the findings for other foundational species, such as corals and sponges^4,46,47^ where there are indirect effects through a food web due to the nutrient provisioning by particular species. Our findings with *P. oceanica* suggest that its role as a host for an active microbiome amplifies its role in these shallow Mediterranean systems.

Ammonification occurred during day and night and with or without *P. oceanica*, suggesting that there is an abundance of microbes that metabolize amino acids and enhance carbon fixation and primary productivity in seagrass ecosystems. Further, every genome that we assembled had the functional capacity for deamination of amino acids to ammonium (Table S6). The use of unfiltered, in situ seawater allowed us to quantify ammonification in the water column, both with and without *P. oceanica*. Bacteria in the water column was likely a significant contributor to ammonification. ^15^N uptake to water column POM was detected in all incubation bottles, though an unknown amount of this uptake could be due to bacteria or eukaryotic phototrophs.

Regardless of whether the ^15^N went into bacteria first, then into water column phytoplankton, the presence of *Posidonia* enhanced the uptake of ^15^N into POM, suggesting that seagrass-associated bacteria, or even fungi, enhance water column productivity both at vent and control sites. The comparison of light versus dark uptake of ^15^N into POM demonstrated that at least some of this uptake can be attributed to bacteria given the continued incorporation of ^15^N into POM at night (Fig. 2d).

One enigmatic aspect of our results is ammonification rates in units of nmol per hour, while ^15^NH_4_ uptake rates could be in umol per hour. We can think of several explanations for this, including that amino acid concentrations that were greater than the 1uM that we assumed would have overestimated enrichment (R_*source*_) and underestimated ammonification in Eq. (1). A second reason is that these processes could be spatially restricted, where microbial production in a biofilm could be immediately followed by host use, causing us to underestimate ammonification in the water column. Thirdly, if amino acid metabolisms are rapid and amino acid concentrations have high flux, we may have underestimated the processing and recycling of nitrogen during incubation. Ammonium concentrations are often not measured to the extent that nitrate concentrations are measured, despite preferential use of the former by macrophytes^48,49^. Yet, our estimates of ammonium uptake by *P. oceanica* during the day from the addition of ^15^N enriched amino acids ranged from 1.17 to 15.33 umol per g dry mass per hour, comparable to the 2.77 umol rate estimated in^50^ for *P. oceanica* in the Mediterranean and suggesting ammonification may contribute to ammonium fluxes and uptake rates here and in other areas.

Estimates of ammonium use and turnover in nature can be high^50^, though bacterial ammonification has been measured only rarely^51^. Also measured rarely is the concentration of DON, including any component parts such as amino acids. Amino acid concentrations have been estimated at 05–1.9 uM in other areas in the Mediterranean^52,53^, but a single flux estimate in the Caribbean suggests the flux could be high^51^. DON could be a persistent source of ammonium, and DON metabolisms warrant increased understanding of their contribution to nitrogen concentrations and fluxes.

Our demonstration of increased δ^15^N in seagrass tissue does not conclusively prove that these plants are taking up ^15^NH_4_ from microbial processes. Only pulse-chase experiments with more precise imaging, for example using NanoSIMS^15,16^, could show that microbial metabolism was the intermediate between ^15^N-amino acids and the higher δ^15^N of macrophyte tissue. However, ammonification genes were ubiquitous across MAGs here (Table S4) and in other studies^14,54^, and suggests that microbes may mediate the availability of ammonium. Several previous studies that suggest seagrasses directly take up DON did not control or account for microbial activity^18^, and thus direct DON uptake by marine angiosperms remains unknown.

Amino acid concentrations in the ocean are rarely reported, though there are several studies in the Mediterranean in proximity to *P. oceanica* beds that report 0.5 to 1.9 uM dissolved free amino acids (DFAA) concentrations^52,53^, several orders of magnitude greater than those reported in open ocean settings^55,56^. DFAA can be a reduced source of nitrogen for both phytoplankton and bacteria^20^, and may be rapidly renewed via the release from animals^57,58^, phytoplankton^59^, and possibly the host themselves, such as *P. oceanica* here^52^. Concentrations can be greater in the sediment surrounding *P. oceanica* when compared to the water column^52^. Although estimates are that DFAA is only from 1 to 10% of DON in coastal areas^60^, the estimates are few and DFAA and DON may have rapid turnover rates which make their contribution to overall nitrogen use difficult to estimate.

*Posidonia oceanica* has other means of acquiring dissolved inorganic nitrogen from microbial associates. Nitrogen fixation genes were found in MAGs from the rhizome at the control site, which is consistent with other studies that have identified nitrogen fixation^61^, *nifH* genes^62^, or nitrogen-fixing taxa in association with *P. oceanica*^15^ in the low-oxygen environment of the rhizomes. Mohr et al.^15^ assembled a MAG for a novel taxon that fixes nitrogen within the cells of *P. oceanica* roots. We did not find the ‘*Candidatus* Celerinatantimonas neptuna’ they described in any of the MAGs we assembled, nor in the family Celerinatantimonadaceae, perhaps due to either incomplete DNA sequencing or to our use of surface swabs rather than grinding the root tissue of *P. oceanica*, as they did. Our analysis of *P. oceanica* blades did not reveal *nifH* genes. Dissimilatory nitrate reduction to ammonium is an additional bacterial metabolism that may help the seagrass host access nitrogen. We found genes for this function in rhizomes in control areas, consistent with previous studies of denitrification in association with seagrass^63^.

Ambient nitrate values were similar between the two sites at the single timepoint when we estimated them; previously published values also cite similar low nitrate concentrations regardless of venting that are < 1 uM^64,65^. Both vent and control sites had microbes that could increase nitrogen to enhance *P. oceanica* primary production. In addition to the abundance of enzymes with deaminating function (Table S4), nitrate reduction genes were indicated, as was nitrogen fixation. Even though estimates of ammonification were greater in control areas, *P. oceanica* carbon fixation did not differ between the control area and the CO_2_ vent area. Carbon fixation rates were similar to the rates estimated at the same location previously^8^ for blades without epiphytes. The NPP values reported by Berlinghof et al. ^8^ in μmol O_2_ convert to ~ 0.7 to 0.9 mg C per g dry mass per hour, assuming a photosynthetic quotient of 1.0 (a 1:1 molar ratio of oxygen release to carbon uptake), a range nearly identical to what we estimated, though they show increased net primary production in proximity to vents. Here, our estimates of primary production included the rhizome tissue which may have obscured carbon fixation differences due to respiration.

There were microbial metabolisms that were unique to *P. oceanica* tissue types, such as cyanobacteria and anoxygenic photosynthesis only on seagrass blades. B vitamin synthesis was also prevalent, but the rhizomes in vent sites lacked Vitamin B1 and B6 synthesis. Vitamin B12, suggested to be auxotrophic for marine eukaryotic hosts^66,67^ was in some MAGs in both tissue types and at both control and vent sites. Whether it plays the critical role in host fitness that has been demonstrated for other marine macrophytes (e.g.^1^) remains to be investigated. In general, alpha diversity and the number of high quality MAGs was low in vent samples. Whether this reflects lower bacterial abundance in these areas is unclear, though certain functions were absent.

Our finding that *P. oceanica* and its microbial associates stimulate water column carbon fixation adds an important dimension to the role of seagrass beds in the carbon cycle. Seagrasses are suggested to alter the dissolved organic nitrogen^51^ and seaweeds can influence water column microbes^68^. As ocean acidification continues, our results suggest that microbial ammonification rates in association with seagrass will decrease. However, the demonstrated role that *P. oceanica* plays in enhancing carbon fixation by surrounding POM, particularly in low pH seawater, suggests an underappreciated role for resilient foundational species in a changing ocean.

## Methods

### Study system

Underwater CO_2_ vent systems are a powerful tool for exploring the effects of low pH on the features of species and biological systems. In Ischia, Italy, CO_2_ vent-influenced *P. oceanica* meadows (Castello South, “vent”, 40.73068047, 13.96303709) are separated by less than a km from areas unaffected by CO_2_ vents and (”control”, Sant' Anna, 40.73053186, 13.96102293). The vent sites at Castello South have a mean pH near 7.7, while the control pH site at Sant' Anna has ambient pH ~ 7.95^28^. Seawater temperature and other environmental variables (e.g. depth, light, salinity) are similar in CO_2_ vent and in control pH sites^28^. While carbon dioxide is the main gas from the vents, other gasses include N_2_ (3.2–6.6%), O_2_ (0.6–0.8%), Ar (0.08–0.1%), and CH_4_ (0.2–0.8%) but not sulfur gas^32^. Trace metal concentrations can be elevated near vents, although detrimental effects on *P. oceanica* have not been recorded^69^.

### ^15^N amino acid incubation with *P. oceanica*

*P. oceanica* was collected in situ at the control pH site at 1000 h and the CO_2_ vent site at 1040 h on 14 Sept 2021. At each site, two blades attached to a single rhizome unit were selected via SCUBA to have both a new blade developing as well as a corresponding older blade that was generally free of epiphytes. The rhizome was removed so that the blades (from 4 to 6) remained within a sheath. *P. oceanica* wet mass was quantified with a Pesola and ranged from 2.0 to 3.5 g (mean = 2.75 g). At each site, 8 *P. oceanica* shoots were collected. While we collected plants that were approximately a half meter distant from each other, we do not know if individuals were genetically distinct.

Simultaneously, gas-tight 500 ml polycarbonate bottles (actual volume is 660 ml) were filled at 1–2 m depth at each site, holding the bottle underwater above the *P. oceanica* beds, filling to eliminate all bubbles, and capping underwater. For each site, *P. oceanica* was immediately added to 8 of the bottles, while 4 held seawater only to quantify water column processes. Oxygen and temperature were recorded immediately with 3 mm optical fiber probe (#OXROB3, Pyroscience, Germany) and temperature sensor (Firesting FS02-4, Pyroscience, Germany) assuming 38 ppt Salinity.

We amended each of the 16 bottles with *P. oceanica* and the 8 with seawater only with amino acids that were enriched in ^15^N (98%, Cambridge Isotope, NLM-2151, Lot#PR-24163). We assumed a concentration of dissolved free amino acids (DFAA) of 1 uM based on previous work in Mediterranean *Posidonia* beds that ranged from 0.5 to 1.9 uM^52,53^. We added 50 uL of a 0.05 M solution to an assumed existing concentration of 1 uM to achieve an enrichment of ~ 0.76 at%. Each bottle was gently inverted 5 times to distribute the isotope, then attached to a line with dive weights at either end and deployed at 2 m depth at noon. All 32 bottles were deployed across 4 lines in the same embayment (Cartaromana Bay), approximately 0.5 km shoreward from where both sets of seagrasses originated, though in waters that were unaffected by the vent activity. The bottles rested on the benthos and they received natural light and some gentle water surge throughout the experiment.

To quantify nitrogen metabolic processes during the daylight versus darkness, we analyzed half of the *P. oceanica* and seawater contained within the bottles after 6 h at 1800 h; the remaining 16 experimental units were removed from the water immediately after sunrise the following day on 15 Sept 2021 (0720 h).

For each of the end of day and end of night censuses, the oxygen concentration in each bottle was measured and seawater was preserved for dissolved organic matter concentration, inorganic nutrient concentrations, including the concentration of ^15^N-ammonium. We also filtered 360–420 ml of seawater through a 0.7 uM gf/f filter (25 mm, Whatman) to quantify the ^15^N of particulate organic matter (POM) in the seawater and dried at 50 °C for 48 h. Dissolved inorganic nutrient samples were filtered through a 0.2 um PE filter and frozen at − 20 °C. Seawater samples for dissolved organic C and N analysis were filtered through pre-combusted GF/F filters into acid-washed HDPE vials, immediately fixed with 160 uL of 18.5% HCl and stored at 4 °C until analysis on a total organic carbon analyzer (TOC-L with TNM-L Unit, Shimadzu Corporation, Japan). DON was obtained from TDN subtracting total dissolved inorganic N (nitrate, nitrite and ammonium), determined on seawater samples preserved frozen and analyzed on a Continuous Flow Analyzer (Flowsys, SYSTEA SpA., Italy).

We analyzed the changes to both organic nutrient (DOC and DON) and inorganic nutrients (nitrate, nitrite, ammonium, phosphorus, silica) when incubation bottles had seawater only or *P. oceanica*, using a univariate three-way ANOVA, followed by two-way ANOVAs with site and treatment and their interaction for both daytime and nighttime.

### Quantifying nitrogen dynamics

We quantified the amino acid mineralization rate (ammonification) using the source-sink model from Lipschultz^70^. The model quantifies the transfer from the source (^15^N amino acids) at the beginning of the experiment to the 'sink', which is the ^15^NH4 in the seawater, either at the end of the day or the end of the night. The rate from *T*_0_ to *T*_*f*_ was based on the following difference equation:

1 $$Amino\, acid\, mineralization\, rate= \frac{{R(t)}_{sink}-{R(t)}_{source}}{\left({R}_{source}-{R\left(0\right)}_{sink}\right)*\mathrm{t}} *\left[\overline{{NH }_{4}}\right]$$

where *R* is the isotopic ratio of the sink or source, t is the length of the incubation, and $\left[\overline{{NH }_{4}}\right]$ represents the average concentration of ammonium over the course of the experiment. By quantifying the metabolism of ^15^N from amino acids (the source) to ammonium in the water column (the sink), we estimated the hourly rate in nmol for the approximately 7 h daytime incubation, as well as the entire 20 h until the next morning. We then separated the ammonification rate for the night only by assuming the *R*(0)_*sink*_ was the mean value at the end of the day. We quantified Eq. (1) for all 8 bottles with unfiltered seawater and 16 incubation bottles with both *P. oceanica* and unfiltered seawater. We used unfiltered seawater from immediately adjacent to the *P. oceanica* used in the experiment to quantify activity by water column versus host-associated microbes at each site.

Because we needed a concentration of ^15^NH_4_ in seawater that we could detect, given the relatively low concentrations of [NH_4_], we added 77.6 μL of 0.05 M NH_4_Cl, effectively adding 1.4 mg/L of NH_4_. The δ^15^NH_4_ value of the NH_4_Cl was known, allowing for reliable detection of ammonium without diluting the ^15^NH_4_ signal^54^.

We tracked the uptake of ^15^N to *P. oceanica* tissue to estimate ammonium uptake rates, following methods in^71^. This method uses the following equation:

2 $${{NH}_{4}}^{+} \, uptake \, rate= \frac{particulate \, 15N \, at\% \, excess}{\left(R*t\right)}*NT$$

where tissue ^15^N atom% excess is the final ^15^N atom% minus the initial ^15^N atom%, *R* is the mean ^15^N atom% enrichment in the NH_4_^+^ pool, *t* is the duration of the incubation, and *N*_*T*_ is the amount of nitrogen in *P. oceanica* in μmols. We also used Eq. (2) to estimate ^15^N uptake by particulate organic matter (POM); for POM the ^15^N could have been in the form of ammonium or amino acids. The possible pathways for the addition of 15N amino acids across our incubations is illustrated in Fig. 1.

The percent carbon, nitrogen and δ^15^N and δ^13^C of *P. oceanica* tissue was measured from a 1 cm section of the midblade where we scraped off all epiphytes, as well as new meristematic tissue at the base that was always free of epiphytes. *P. oceanica* tissue was sampled prior to enrichment, at the end of the daylight hours, and following the nighttime period. The tissue was dried, ground to a fine powder in a GenoGrinder Spex (Metuchen, NJ), and analyzed on an elemental-analyzer–isotope-ratio mass spectrometer at Northwestern University Stable Isotope Biogeochemistry Laboratory (NUSIBL).

All statistical analyses were performed in R (www.R-project.org), version 4.2.2 (2022-10-31). For rate estimates that were highly variable and non-zero, we logged to reduce variance, prior to ANOVA.

### Metagenome analyses

The surface microbiome of *P. oceanica* was sampled with a sterile cotton swab on the upper rhizome tissue; the mid blade region (corresponding to 2 above) and the older tissue with epiphytes (3. above) was sampled with both a cotton swab and Dentek brush to ensure a complete surface sample on blade tissue. All were placed in − 20 °C and shipped to storage at − 80° C, then shipped frozen to the University of Chicago. DNA was extracted with a Qiagen PowerSoil Kit and multiple samples were pooled for each of 6 metagenome samples to increase DNA quantity and possible discovery. The concentration of DNA was less on vent site samples compared with the control site, so we pooled all 6 sampled individuals into a single sample for each of the vent blade and vent rhizome. Thus, *P. oceanica* blade samples were pooled from 6 plants at the vent site and 3 plants each at control site; rhizome samples were also pooled from 6 plants at the vent site and 3 plants each at the control site. We thus had duplicated samples for control blades and rhizomes but only a single sample for vent tissue type.

The above 6 samples were run over 1 lane on a HiSeq 2500 (2 × 150) with TruSeq DNA library preps at Argonne National Laboratory. For each sample, resulting DNA sequences were first quality filtered^72^, then assembled with IDBA-UD v1.1.3^73^ with a minimum scaffold length of 1 kbp. Metagenomic short reads from each sample were then recruited back to their corresponding assembled contigs using Bowtie2^74^. Samtools^75^ was used to generate sorted and indexed BAM files. Anvi’o v7.0^76^ was used as the command line environment for all downstream analyses. ‘anvi-gen-contigs-database’ was used to generate anvi’o contigs databases, during which Prodigal v2.6.3^77^ identified open reading frames, and ‘anvi-run-hmms’ was used to identify genes matching to archaeal and bacterial single-copy core gene collections using HMMER^78^. We quantified overall alpha diversity with 'anvi-estimate-scg-taxonomy'. We then reconstructed genomes from the assembled metagenomes, we used a combination of automatic binning via CONCOCT v1.1.0^79^, followed by a manual curation of each MAG that had greater than 50% completion, following guidance by^80^ Genome taxonomy was determined using the GTDB (v.1.3.0,^81^) database and 'anvi-run-scg-taxonomy'. We also inferred gene-level taxonomy using Centrifuge v1.0.4^82^ to aid manual curation.

We examined the metabolic capability of the bacterial MAGs associated with *P. oceanica* using ‘anvi-estimate-metabolism’ on the manually refined bins that were > 50% complete and < 10% redundancy^36^. We classified a metabolic module as complete if 75% percent of the genes within the module were complete.

We further quantified the presence of genes that could increase host access to nitrogen. Our experiment specifically tested whether amino acid deamination occurred, so we searched within our assembled MAGs for ammonification hydrolases, including ureases and ammonia-lyases that cleave the C–N bonds in amino acids and make ammonium available to the host. We identified these enzymes through the Enzyme Commission numbers, and searched for EC numbers classified as acting on CH–NH_2_ bonds (EC:1.4.*), acting on carbon–nitrogen bonds other than peptides (EC:3.5.*), or ammonia lyases (EC:4.3.1*), where * indicates any subset of these classifications, and determined whether a MAG had each of these 3 classes of enzymes (e.g.^14^). The Kegg Orthologs^83^ associated with each of these EC numbers is shown in Supplemental Table 5. We searched for evidence of nitrogen fixation by assessing the completion of module 00175, as well as other nitrogen cycling genes and functions that could benefit the host.

All methods on the marine angiosperm, *Posidonia oceanica*, were carried out in situ and in accordance with local and national guidelines.

### Supplementary Information

Supplementary Tables.

Supplementary Figures.

## Data Availability

Environmental data have been archived at Zenodo, https://doi.org/10.5281/zenodo.7834043;metagenomic data are archived at NCBI, http://www.ncbi.nlm.nih.gov/bioproject/967736 and at Figshare: https://figshare.com/collections/Posidonia_oceanica_metagenomics_and_ammonification_Scientific_Reports/6918289.
