# Supplementary material for: Microbial associates of an endemic Mediterranean seagrass enhance the access of the host and the surrounding seawater to inorganic nitrogen under ocean acidification
Source: Sci Rep. 2023 Nov 15;13:19996. doi: 10.1038/s41598-023-47126-4 (PMC10651887; doi:10.1038/s41598-023-47126-4)
Supplement: Supplementary file 2 — Supplementary Figures. [file 41598_2023_47126_MOESM2_ESM.docx]

**Supplementary Figures**


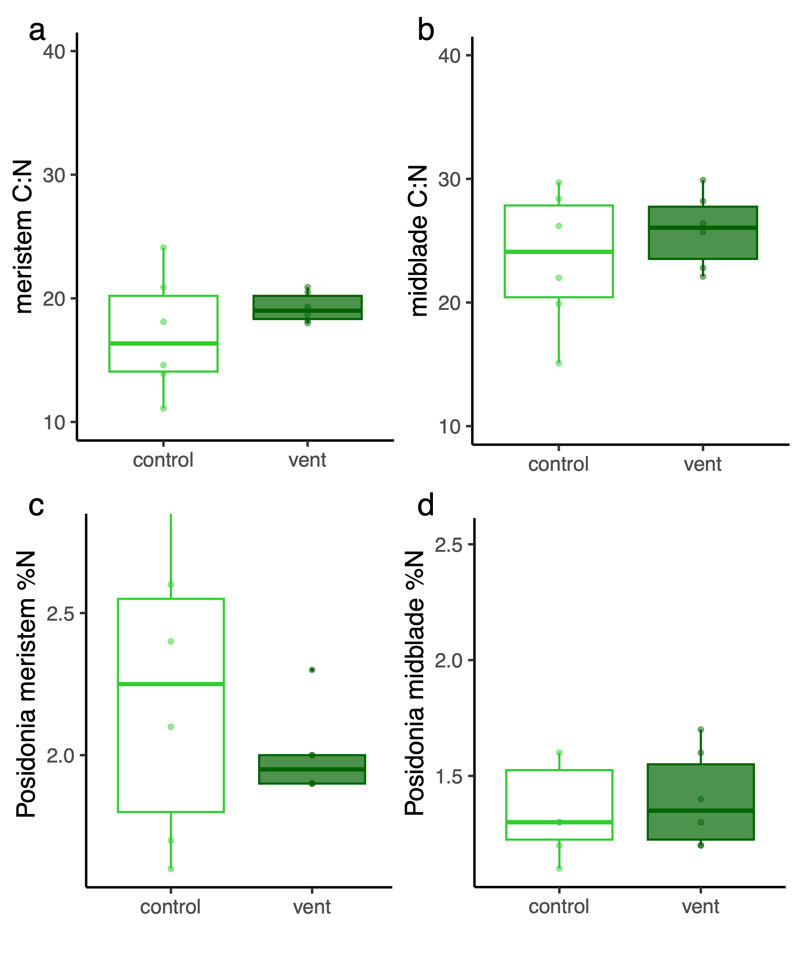


Supplementary Figure 1. *P. oceanica* tissue and POM carbon and nitrogen content. The ratio of tissue carbon to nitrogen did not differ in the a. meristem (n=6, t=1.057, p = 0.334) or b. the midblade region (t = 0.888, p= 0.401), nor were there differences in the overall nitrogen content for either c. meristem or d. midblade. Data and statistical comparisons are summarized in Table S3.


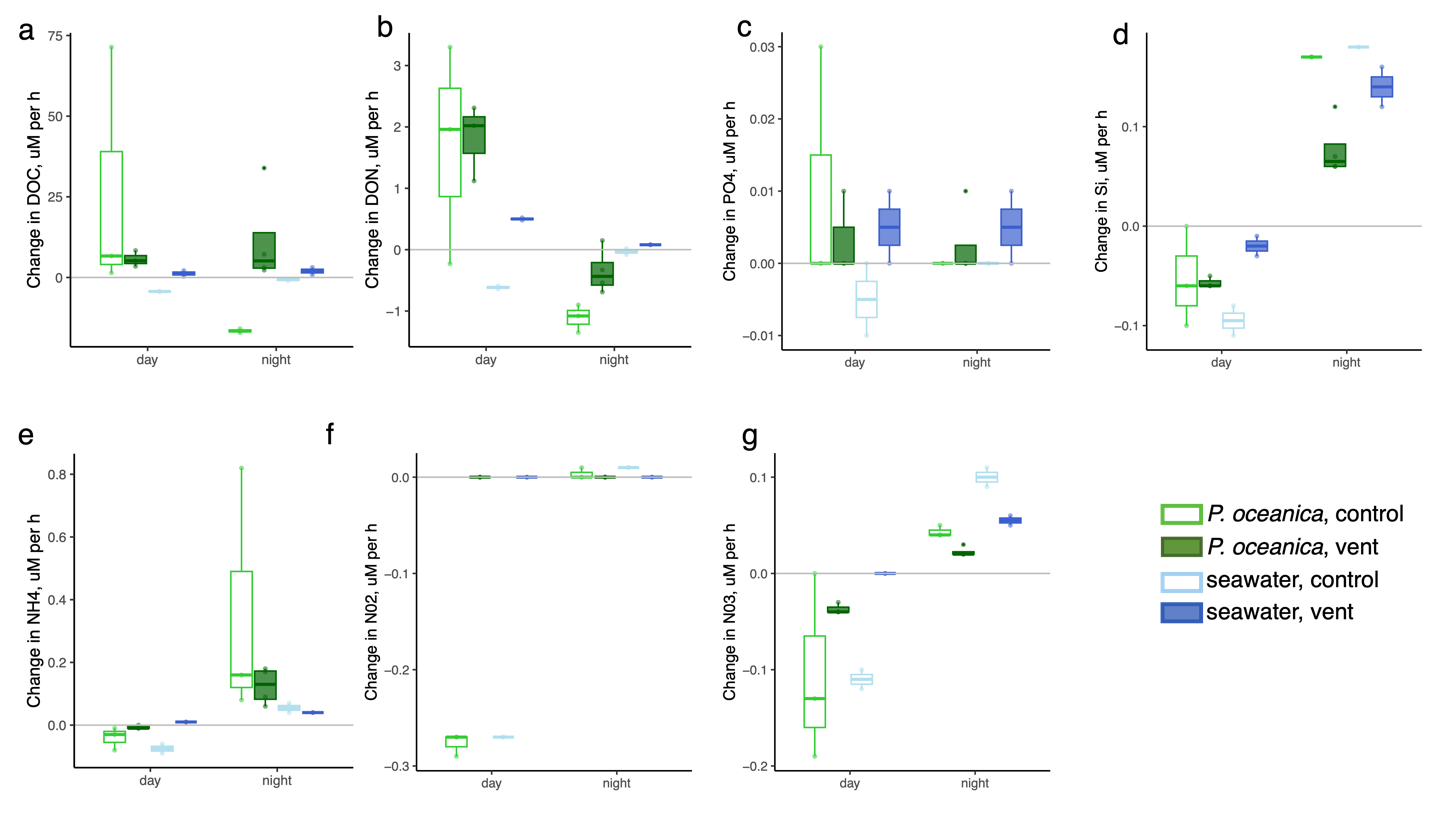


Supplementary Figure 2. Patterns of organic and inorganic nutrients in the incubations throughout the experiment, quantified as the change in uM per hour. The horizontal gray line represents no change. Initial values are in Table S1 and Table S2 shows the ANOVA results grouped by day and night.

Supplementary Tables 1-7 are combined in a single Excel File
